# 

**Published:** 1971-04

**Authors:** 


					Bristol Medico-Chirurgical Journal Vol. 86 (ii) No. 318
Contents
Dermatoglyphics in Medicine
T. J. David
19
Assessment Unit for the Mentally Retarded
J. Jancar
27
Decompression of the Carpal Tunnel
J. E. Woodyard
33
Obituary
37
Wisdom from the Past
38
Society Reports
39
The Medical Library     41
News and Notes ... ... ??? ??? ??? ??? 42
F. Bailey & Son Ltd., Dursley, Glos.

				

